# Interprofessional Collaboration to Address Elder Abuse, Neglect, and Exploitation With Adult Protective Services

**DOI:** 10.1093/geroni/igaa057.2435

**Published:** 2020-12-16

**Authors:** Pi-Ju Liu, Bridget Penhale

## Abstract

Adult Protective Services (APS) is responsible for investigating reports of abuse, exploitation, and neglect among vulnerable adults. Additionally, APS also refers or provides needed services to victims. Though APS also takes the lead position in investigation and service planning, determining harm and designing person-centered remedies often require other professionals to be prepared to address these elder justice issues with APS. Afterall, adult safeguarding is everybody’s business. This symposium includes four presentations on researchers’ findings working with APS and other professionals. Dr. Burnes will compare outcomes on usual APS care (APS only) versus enhanced APS care (APS plus advocate who are case managers). Dr. Pi-Ju (Marian) Liu will examine nurses’ role in working in and with APS, focusing on how their job responsibilities are different from other nurses and APS social workers. Dr. Jason Burnett will present an innovative referral portal linking banks and other financial firms directly to APS. Lastly, Dr. Zachary Gassoumis will appraise the multidisciplinary teams across the country, including professionals involved, and provide input on what makes a team work well together. Following the four presentations, Dr. Bridget Penhale will open up the discussion regarding protective agencies’ operations and collaboration with other agencies, focusing on the comparison between protective agencies in the United States and European countries. Abuse, Neglect and Exploitation of Elderly People Interest Group Sponsored Symposium.

